# Evolution of Procedure Indication and Cardiovascular Risk in Transvenous Lead Extraction

**DOI:** 10.3390/jcm11133596

**Published:** 2022-06-22

**Authors:** Daniel Hofer, Michelle C. Bebié, Noah Kuster, Jan Steffel, Alexander Breitenstein

**Affiliations:** Division of Electrophysiology, Department of Cardiology, University Heart Center, University Hospital Zurich, Raemistrasse 100, 8091 Zurich, Switzerland; michelle.bebie@uzh.ch (M.C.B.); noah.kuster@icloud.com (N.K.); jan.steffel@hirslanden.ch (J.S.); alexander.breitenstein@usz.ch (A.B.)

**Keywords:** transvenous lead extraction, indication, cardiovascular risk, risk factors

## Abstract

**Background:** The use of cardiac implantable electronic devices (CIEDs) to treat tachy- and bradyarrhythmia has significantly increased over the past decades. Consequently, transvenous lead extractions (TLE) have been performed more frequently, particularly in the treatment of device infection or malfunction. We aimed to evaluate the development of procedure indications and cardiovascular risk factors of patients undergoing TLE over time. **Materials** and methods: 277 TLE cases from 2013 to 2020 performed at the University Hospital Zurich were included in this retrospective analysis. Patient charts and follow-up letters were screened for procedure indication and cardiovascular risk factors to evaluate trends over time. **Results:** 502 leads were extracted in 273 patients. The main indications for TLE remained lead dysfunction (48.7%) and infection (31.4%) throughout the investigated period; however, infections were less and device upgrade more frequently encountered indications for TLE over time. Mean patient age at the time of TLE (64.0 ± 0.9 in the entire sample) decreased over time, while the incidence of chronic kidney disease (33.6%), heart failure (48.6%), or diabetes mellitus (22%) demonstrated an increasing trend. **Conclusions:** The main indications for TLE remain device malfunction and infection, while device upgrade was increasingly encountered as an indication for TLE in recent years. Over time, patients undergoing TLE were increasingly younger and more often presented with cardiovascular risk factors.

## 1. Introduction

Cardiac implantable electronic devices (CIEDs) have increasingly been used for the treatment and prevention of cardiac arrhythmias throughout the past years [1,2,3,4]. Consequently, transvenous lead extractions (TLE) have increasingly been performed, mainly to maintain device functionality in case of malfunctions or removal of the source of bacteremia in the case of device infection [5]. TLE has increasingly been performed at our institution over the past decade, amounting to 79 extractions performed in 2020. While the number of TLE performed is increasing from year to year, little is known about the changes in procedure indications and cardiovascular risk factors over time. Whether the cardiovascular risk-profile of patients undergoing TLE has changed with TLE being increasingly performed is unclear. Similarly, whether more patients with the same indications have their leads removed, or if indications have shifted to new patient groups, is unclear. In this retrospective analysis of TLE procedures performed at our institution, we aimed to evaluate the temporal evolution of procedure indications and cardiovascular risk factors over time.

## 2. Materials and Methods

All patients undergoing percutaneous transvenous lead extraction of CIED at the University Hospital Zurich between 2013 and 2020 were included in this retrospective analysis. Every TLE procedure was considered as a separate entity, without regards to patient identity in order to allow for multiple TLE within the same patient to be included in the study, as long as the second TLE procedure was not performed during the same hospital stay. Lead revision for leads implanted less than one year prior to removal were considered explantations and were hence excluded from the analysis. Patient data was collected anonymously. The indications for CIED implantation and transvenous extraction were gathered from the procedure reports of TLE interventions. Patient age, left ventricular ejection fraction (LVEF), arterial hypertension, chronic kidney disease, and diabetes mellitus were gathered from patient charts during the time of TLE. The temporal evolution of TLE indication, each cardiovascular risk factor (CVRF) and the presence or combined presence/absence of CVRF was examined over the investigated eight-year period. Mean values were calculated relative to the entire patient sample and additionally calculated on a year-to-year basis. For visual representation of data trends over time, years were split into terciles: T1 (1 January–30 April), T2 (1 May–31 August), or T3 (1 September–31 December). Exceptions were made in cases where the data was too sparse and results were consequently summarized on a per-year basis. Continuous variables are expressed as mean ± standard error of the mean, while categorical variables are expressed as absolute numbers and percentages. Results were plotted in dot diagrams including a trendline by method of ordinary least square regression analysis for better visual illustration of data developments over time.

## 3. Results

### 3.1. Procedure Indications

**Figure 1:**
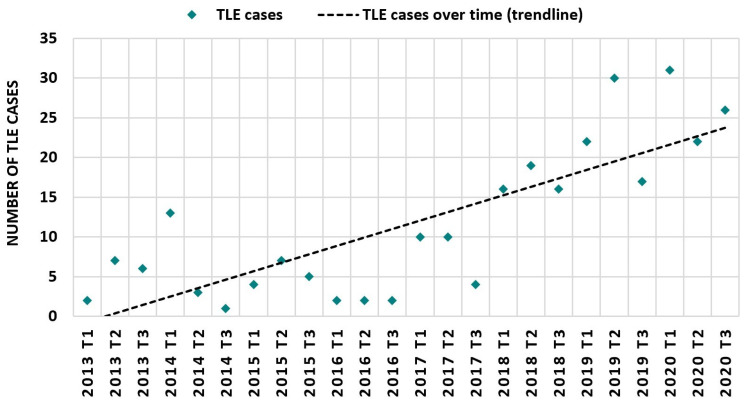
Number of TLE cases over time. The number of TLE cases performed at the University Hospital Zurich is depicted for each four-month period. TLE: transvenous lead extraction.

**Figure 2:**
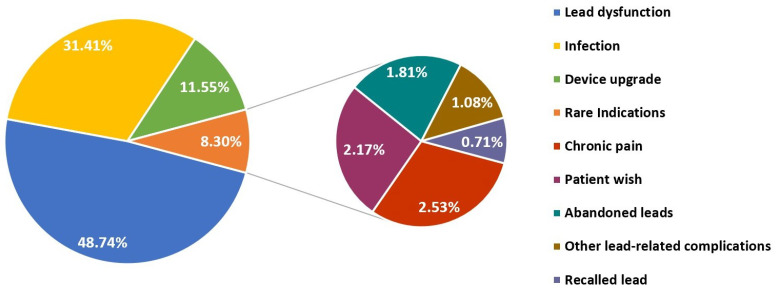
Indications for transvenous lead extraction. Distribution of indications for transvenous lead extraction between 2013 and 2020. The most common indications are illustrated on the left (lead dysfunction, infection, device upgrade), while rare indications are further detailed on the right.

A total of 502 device leads were extracted during 277 TLE procedures on 273 patients, performed between 1 January 2013 and 31 December 2020, cumulating in 1.8 ± 0.1 (range 1–5 leads) leads removed per procedure. In four patients, TLE was performed twice during the study period (three for recurring lead defects, one for additional extraction with jugular snare approach). TLE was performed by three operators during the study period, with one single operator performing 74% of all procedures. The number of TLE procedures increased from two in T1 of 2013 to 26 in T3 of 2020, or, in total, from 15 procedures in 2013 to 79 procedures in 2020 (Figure 1). Lead dysfunction in the setting of device malfunction (48.7%) and infection (31.4%) were the main indications for TLE (Figure 2). Infections warranting TLE were either classified as systemic (63.2%) or in fewer cases considered as local pocket infections (36.8%). TLE was performed less frequently because of a planned device upgrade (11.6%), and in 8.3% other rare indications dictated TLE. Previously abandoned leads rarely served as primary indication for lead extraction (1.8%), although extraction of abandoned leads or lead fragments was attempted in the course of a lead extraction procedure based on other indications in an additional 13 interventions (4.7% of the entire sample).

### 3.2. Evolution of Procedure Indications over Time

**Figure 3:**
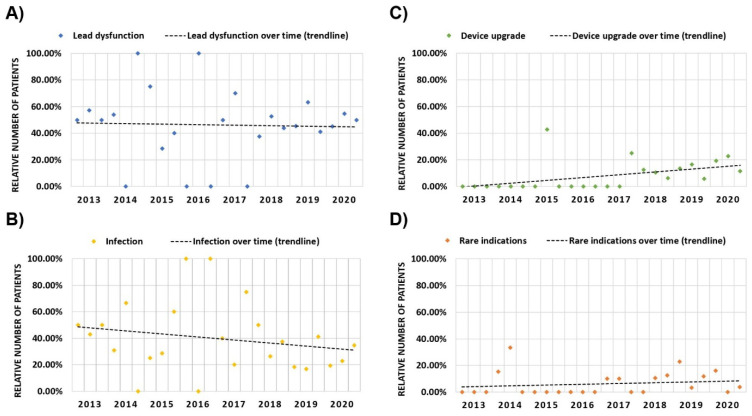
TLE indications over time. The relative number of patients requiring TLE due to lead dysfunction (**A**), infection (**B**), device upgrade (**C**), and rare indications (**D**) over time. Data is demonstrated for every four-month period including trendline analysis.

Lead dysfunction remained the most prominent indication throughout the observed time period at a relatively stable percentage of around 50% of all indications for TLE in trendline analysis, with only a minor decrease over time (Figure 3). In contrast, the trendline analysis demonstrated a reduction in the percentage of patients undergoing TLE due to infection over time (Figure 3), while device upgrades increased as an indication for TLE over time (Figure 3). While rarely mentioned as indication for TLE prior to 2017, device upgrade has been listed as the indication for TLE between 10–20% of all interventions in the most recent four-month periods. Similarly, rare indications seem to demonstrate a slight increase in our trendline analysis over time (Figure 3).

### 3.3. Cardiovascular Risk Factors

Patients were predominantly male in 73.3% (26.7% female) and the mean patient age at the time of extraction was 64.0 ± 0.9 years (range 19 to 94 years). A total of 54.5% of patients had arterial hypertension, 22% had diabetes mellitus, and 33.6% had chronic kidney disease. Mean LVEF at the time of the procedure was 45.2 ± 0.9 (range 10% to 79%) with 48.6% and 30.4% of patients demonstrating an LVEF < 50% and ≤35%, respectively.

### 3.4. Evolution of Cardiovascular Risk Factors over Time

**Figure 4:**
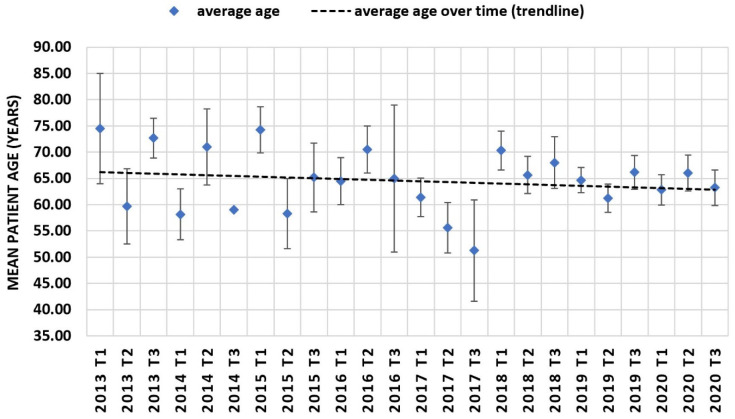
Mean patient age at TLE. Mean patient age at extraction including standard error of the mean for every four-month period and trendline analysis. Only a single patient was recorded for 2014 T3; therefore it is without standard error of the mean.

**Figure 5:**
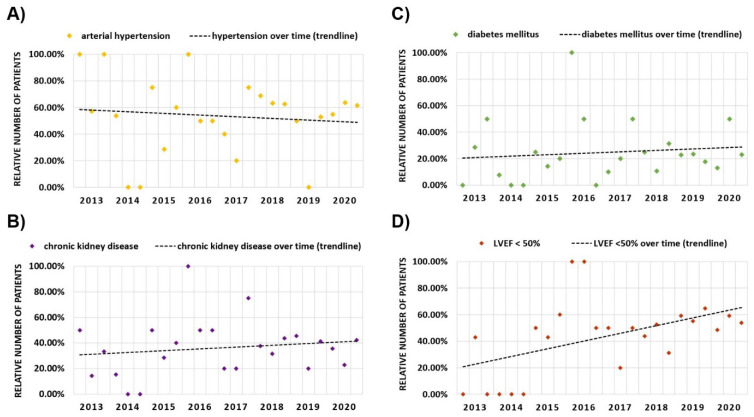
Cardiovascular risk factors over time. The relative number of patients diagnosed with arterial hypertension (**A**), chronic kidney disease (**B**), diabetes mellitus (**C**), and LVEF < 50% (**D**) prior to transvenous lead extraction over time. Data is demonstrated for every four-month period including trendline analysis. LVEF: left ventricular ejection fraction.

**Figure 6:**
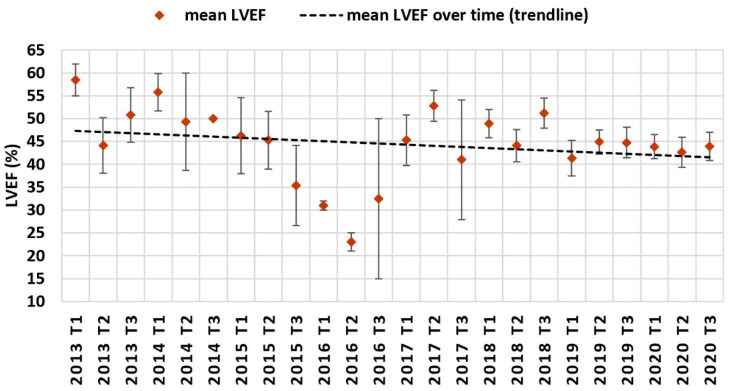
Mean left ventricular ejection fraction over time.

**Figure 7:**
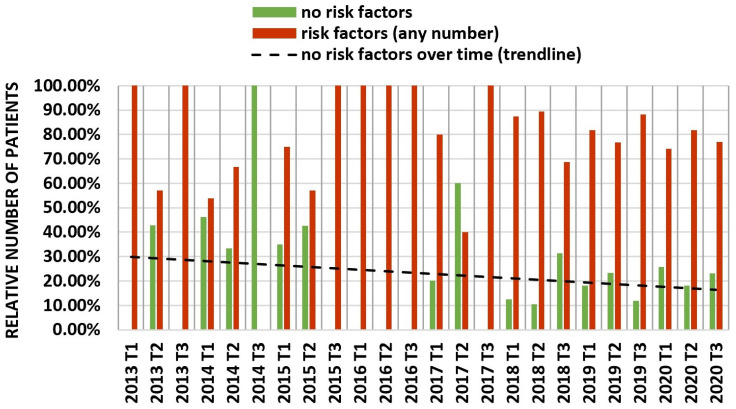
Presence or absence of cardiovascular risk factors. The percentage of patients without any cardiovascular risk factor (left ventricular ejection fraction < 50%, arterial hypertension, diabetes mellitus, chronic kidney disease) is compared to the percentage of patients with ≥1 cardiovascular risk factor for every four-month period over time including trendline analysis.

Trendline regression analysis demonstrated decreasing age at TLE over time with increased variability in patient age in earlier years due to lower sample sizes per time period (Figure 4). Concerning other cardiovascular risk factors, trendline analysis demonstrated a decreasing percentage of patients diagnosed with arterial hypertension, but an increasing amount of patients diagnosed with chronic kidney disease, diabetes mellitus and heart failure (Figure 5). For heart failure, mean LVEF at time of TLE (Figure 6) also demonstrated a decreasing trend. To evaluate the combined cardiovascular risk profile of patients undergoing TLE over time, the percentage of patients with and without the predefined risk factors was plotted for every four-month period over time (Figure 7). A trendline analysis demonstrated a declining number of patients undergoing TLE without any of the studied risk factors (arterial hypertension, diabetes mellitus, chronic kidney disease, LVEF < 50%) over time. In every four-month time period, at least 40% of all patients undergoing TLE demonstrated at least one CVRF.

## 4. Discussion

### 4.1. Procedure Indication for TLE

In our retrospective single-center cohort study, lead dysfunction remained the most frequent indication for TLE at around 50% of all procedures over the entire study time period. The decreasing trend of infection as indication for TLE is somewhat surprising, since the percentages of device infections after CIED implantation have been increasing in previous studies [6,7]. Interestingly, device upgrade and other rare indications appear as an increasing indication for TLE over time and may partially explain the increasing amount of TLE performed overall as well as the relative decrease in infection as indication for TLE. However, lead dysfunction remained stable over time, suggesting either a less pronounced increase in this indication compared to device upgrade and rare indications over time, or in fact a true decrease in infection as indication for TLE. Increasing operator- and center experience over time may have facilitated decisions regarding abandonment versus extraction of old leads at the time of a planned device upgrade. In the same way, an increasing amount of referrals for TLE with rare indications may provide an explanation.

### 4.2. Cardiovascular Risk at the Time of TLE

Our retrospective single-center cohort study demonstrated that patients undergoing TLE seem to become younger and present increasingly with cardiovascular risk factors such as diabetes mellitus, chronic kidney disease, or heart failure at the time of TLE. This may be explained by an increasing use of CIED in younger and sicker patients [2]; alternatively, this may have resulted from increasing operator and center experience over time positively influencing the procedure’s risk–benefit ratio thereby influencing the decision to perform TLE in younger and sicker patients. Additionally, the incidence of cardiovascular risk factors including diabetes mellitus and chronic kidney disease is increasing in Switzerland in the general population, which may also explain these findings [8,9,10,11,12]. Since three of the four cardiovascular risk factors in our study demonstrated increasing trends, the percentage of TLE patients without any cardiovascular risk factors declined overall during the observed time period. Furthermore, mean LVEF before TLE declined throughout the study period. In summary, these observations demonstrate an overall increase in the pre-operative risk of TLE patients at the time of the procedure.

### 4.3. Limitations

The single center and retrospective design, referral bias, and selection bias, are the main limitations of this study. By virtue of the design of our study, no causal relationships may be inferred and only associations are reported. Trend analysis for CVRF and indications for TLE all demonstrated presumed outliers with consequent possible distortion of the analysis. For a coherent statistical analysis of significance, larger multi-center registries should be consulted.

## 5. Conclusions

The main procedure indication for TLE remains lead dysfunction and infection, with device upgrade presenting an increasingly frequent indication in recent years. Patients undergoing TLE present with a younger age and more cardiovascular risk factors in recent years.

## Data Availability

Upon urgent request and associated need, our data is available, while our utmost intention is to protect our patient’s privacy.

## Abbreviations

CIED	Cardiac implantable electronic device
TLE	Transvenous lead extraction
LVEF	Left ventricular ejection fraction
CVRF	Cardiovascular risk factors
